# Use of SGLT2 inhibitors and GLP-1 receptor agonists in patients with ischaemic heart disease and type 2 diabetes in Swedish primary care: a cross-sectional analysis of regional primary care registry data (QregPV)

**DOI:** 10.1136/bmjopen-2025-110395

**Published:** 2026-02-02

**Authors:** Tobias Andersson, Johan-Emil Bager, Margareta Hellgren, Maria Åberg, Georgios Mourtzinis

**Affiliations:** 1 General Practice/Family Medicine, School of Public Health and Community Medicine, Institute of Medicine, Sahlgrenska Academy, University of Gothenburg, Gothenburg, Sweden; 2 Regionhälsan R&D Centre, Skaraborg Primary Care, Skövde, Sweden; 3 Department of Molecular and Clinical Medicine, Institute of Medicine, Sahlgrenska Academy, University of Gothenburg, Gothenburg, Sweden; 4 Department of Emergency Medicine, Sahlgrenska University Hospital, Gothenburg, Sweden; 5 Regionhälsan, Region Västra Götaland, Gothenburg, Sweden; 6 Department of Medicine, Geriatrics and Emergency Care Mölndal, Sahlgrenska University Hospital, Gothenburg, Sweden

**Keywords:** Diabetes Mellitus, Type 2, Primary Health Care, Guideline Adherence, Drug Therapy, Coronary heart disease

## Abstract

**Abstract:**

**Objectives:**

To assess the use of sodium-glucose cotransporter 2 inhibitors (SGLT2i) and glucagon-like peptide 1 receptor agonists (GLP-1 RA) among patients with coexisting ischaemic heart disease (IHD) and type 2 diabetes (T2D) in primary care, in relation to European guidelines.

**Design:**

Cross-sectional observational study.

**Setting:**

209 primary healthcare centres in Region Västra Götaland, Sweden (population 1.8 million in 2023).

**Participants:**

14 414 patients with registered prevalent diagnoses of coexisting IHD and T2D, September 2023, in QregPV, the regional primary care quality of care register in Region Västra Götaland. Data on dispensed drugs were retrieved from the regional prescribed drug register, Digitalis.

**Primary and secondary outcome measures:**

The primary outcome was the proportion of patients with dispensed SGLT2i or GLP-1 RA in relation to sex, age and primary healthcare centres (including private vs public ownership). The secondary outcome was estimated additional prescription costs.

**Results:**

SGLT2i was dispensed to 37.2%, less often to women (adjusted OR (aOR) 0.64 (95% CI 0.59 to 0.70)). GLP-1 RA was dispensed to 10.0%, with no sex difference (aOR 1.04 (95% CI 0.92 to 1.18)). Use of SGLT2i and GLP-1 RA declined with age (p<0.001). Use across primary healthcare centres (95% central range) varied from 17.1% to 56.4% for SGLT2i and 0.0% to 23.4% for GLP-1 RA, without differences between private versus public primary healthcare centres (SGLT2i: aOR 0.95 (95% CI 0.85 to 1.06); GLP-1 RA: aOR 1.06 (95% CI 0.89 to 1.26)). Variation across primary healthcare centres was substantial (SGLT2i: adjusted median OR (aMOR) 1.29 (95% CI 1.23 to 1.36); GLP-1 RA: aMOR 1.48 (95% CI 1.37 to 1.62)). Treating all patients would increase the annual prescription costs, €3.9 million for SGLT2i and €10.4 million for GLP-1 RA.

**Conclusion:**

SGLT2i and GLP-1 RA were underutilised in patients with coexisting IHD and T2D. The sex disparity in SGLT2i use warrants attention, as does the substantial variation between primary healthcare centres and the challenges of implementing costly cardioprotective therapies.

**STRENGTHS AND LIMITATIONS OF THIS STUDY**

- The study included 99.5% of all primary healthcare centres in the region, ensuring high internal validity.
- Variation in use of sodium-glucose cotransporter 2 inhibitors (SGLT2i) and glucagon-like peptide 1 receptor agonists (GLP-1 RA) was assessed across primary healthcare centres.
- Individual-level data on dispensed SGLT2i and GLP-1 RA, more accurately reflecting patients’ actual use than prescribed medication data.
- Limited data were available on comorbidities and individual socioeconomic factors.
- Variability in drug pricing and reimbursement policies may affect the generalisability of the findings to other healthcare settings.

## Introduction

 In type 2 diabetes (T2D) with or without concomitant ischaemic heart disease (IHD), the first-line choice for blood glucose-lowering medication has long been treatment with metformin. Since newer but also more expensive drugs, sodium-glucose cotransporter 2 inhibitors (SGLT2i) and glucagon-like peptide 1 receptor agonists (GLP-1 RA) have been shown to not only decrease blood glucose but also reduce the risk of cardiovascular (CV) morbidity and mortality, they are recommended in European Society of Cardiology (ESC) guidelines as first-line drugs for T2D in combination with IHD, before metformin and independent of blood glucose levels.^1 2^

In Sweden, the downgrading of metformin has not yet been fully reflected in clinical guidelines, where SGLT2i and/or GLP-1 RA are recommended in addition to metformin. However, increased use of SGLT2i and GLP-1 RA has been seen in patients under 80 years of age with T2D who have recently experienced a myocardial infarction.^3^

Low utilisation of SGLT2i and GLP-1 RA has been reported among patients in primary care with IHD and newly diagnosed T2D.^4^ However, less is known about patients with a heterogeneous duration of disease. Moreover, little is known about the usage of SGLT2i and GLP-1 RA in primary care among patients with IHD and T2D, or how it varies between primary healthcare centres (PHCCs) and by public versus private ownership. Studies of veterans in the USA have shown underuse of SGLT2i and GLP-1 RA in patients with IHD and T2D, with and without chronic kidney disease, with significant variation in usage between different healthcare facilities.^5^,^7^ A high level of co-payment for patients has been associated with a lower degree of adherence to SGLT2i and GLP-1 RA in heart failure and T2D.^8^ In Sweden, the cost of healthcare visits and prescribed medications is universally subsidised by the government with low out-of-pocket costs for the individual patient, potentially affecting the usage of SGLT2i and GLP-1 RA. Given the CV risk reduction associated with SGLT2i and GLP-1 RA, insufficient implementation may represent a missed opportunity for secondary prevention in a large and high-risk patient population.

The aim of this study was to explore the usage of SGLT2i and GLP-1 RA in primary care among patients with IHD and T2D, and how it varies according to age, sex and PHCCs, including whether public versus private ownership. We also aimed to estimate the potential additional cost for prescribed SGLT2i and GLP-1 RA if used according to ESC guidelines.

## Methods

### Study design and setting

This cross-sectional observational study included patients registered in QregPV, a primary care quality of care register in Region Västra Götaland (VGR), the second largest county in Sweden, with a mixed urban and rural population of 1.77 million inhabitants in August 2023.

### Participants and data sources

The study included patients with concomitant diagnoses of IHD (International Classification of Diseases 10 (ICD-10): I20–I25) and diabetes (ICD-10: E10–E14) registered in QregPV as of 1 September 2023 (index date). As previously described, all approximately 210 PHCCs in VGR report monthly clinical data to QregPV regarding patients with a diagnosis of IHD, diabetes, hypertension, chronic obstructive pulmonary disease (COPD) and asthma.^9^ As patients with type 1 diabetes are not primarily managed in primary care and 98% of patients with diabetes in Swedish primary care reported to the National Diabetes Register are classified as having T2D,^10^ individuals with a recorded diagnosis of diabetes in QregPV are, in practice, considered to have T2D and are referred to as such throughout this study. Details on registered diagnoses and clinical data in QregPV are presented in online supplemental table S1. For each PHCC, administrative data from VGR were added regarding burden of disease (adjusted clinical group, ACG),^11^ socioeconomic status (care need index, CNI),^12^ number of enrolled patients and whether public or private ownership. ACG is a method for describing patient case-mix based on age, sex and registered diagnoses to predict healthcare utilisation. CNI is based on sociodemographic factors reflecting social deprivation (elderly persons living alone, foreign-born individuals, unemployed individuals, single parents, high residential mobility, individuals with low educational status and children under the age of 5).

In Sweden, dispensed drugs are registered in the Prescribed Drug Register^13^ and in VGR, they are registered in parallel in the regional prescribed drug register Digitalis. Using the Swedish unique personal identification number, which is issued to all inhabitants at immigration or birth, data on dispensed drugs from Digitalis up to 1 year before 1 September 2023 were retrieved for all study participants (online supplemental table S2).

### Use of SGLT2i and GLP-1 RA

Medications in Sweden are usually prescribed for 90–100 days with multiple fills per prescription. The study outcomes were dispensed SGLT2i (empagliflozin, dapagliflozin, canagliflozin) and GLP-1 RA (semaglutide, dulaglutide, liraglutide, lixisenatide) during the last 120 days before the index date (1 September 2023). In sensitivity analyses, dispensed medications up to 1 year before the index date were analysed. The proportion of patients who were dispensed these drugs was assessed according to sex, age and different PHCCs including whether public versus private ownership. Annual out-of-pocket costs at the pharmacy for treatment with SGLT2i and GLP-1 RA in 2023 were obtained from official data provided by the Swedish Dental and Pharmaceutical Benefits Agency (TLV).^14^

### Statistical analysis

Descriptive statistics were used to present patient characteristics and dispensed drugs, categorised by sex and age groups. The proportions of patients with dispensed SGLT2i or GLP-1 RA were estimated with 95% CIs. Logistic regression models, adjusted for age as a continuous variable, were used to test differences in drug dispensation by sex, with robust 95% CI accounting for clustering at the PHCC level. Similarly, differences in dispensation according to privately versus publicly owned PHCCs were tested, adjusting for sex and age.

The proportions of patients receiving SGLT2i and GLP-1 RA were ranked and plotted for each PHCC. The distribution of these proportions was described using the median and the 2.5th and 97.5th percentiles. To assess heterogeneity in dispensed drugs at the PHCC level, multilevel regression models were used to estimate the median OR (MOR) with 95% CI.^15^ First, an empty model, containing only PHCC-specific random effects, was used to estimate the MOR. The second model adjusted for patient characteristics (age, sex and the other diagnoses available in QregPV: hypertension, asthma or COPD). A third model further adjusted for PHCC characteristics (CNI, ACG, number of enrolled patients and ownership type). An MOR of, for example, 1.50 indicates that, among two identical (conditional on covariates) patients at two randomly selected PHCCs, the odds are 50% higher of receiving the drug at the PHCC with the higher dispensing rate. Sex, hypertension, asthma, COPD and ownership were treated as binary variables, while age, CNI, ACG and patient numbers were continuous and scaled. In a sensitivity analysis, natural splines were used for continuous variables to account for non-linearity. Direct standardised rates of SGLT2i and GLP-1 RA usage were estimated and plotted for each PHCC using multilevel models, with the 2023 VGR patient population as the reference. All analyses were performed using complete cases. Statistical tests were conducted at a 0.05 significance level and were two-tailed. Analyses were performed using R V.4.3.3 and RStudio V.2024.12.1.^16 17^

### Patient and public involvement

None.

## Results

**Figure 1:**
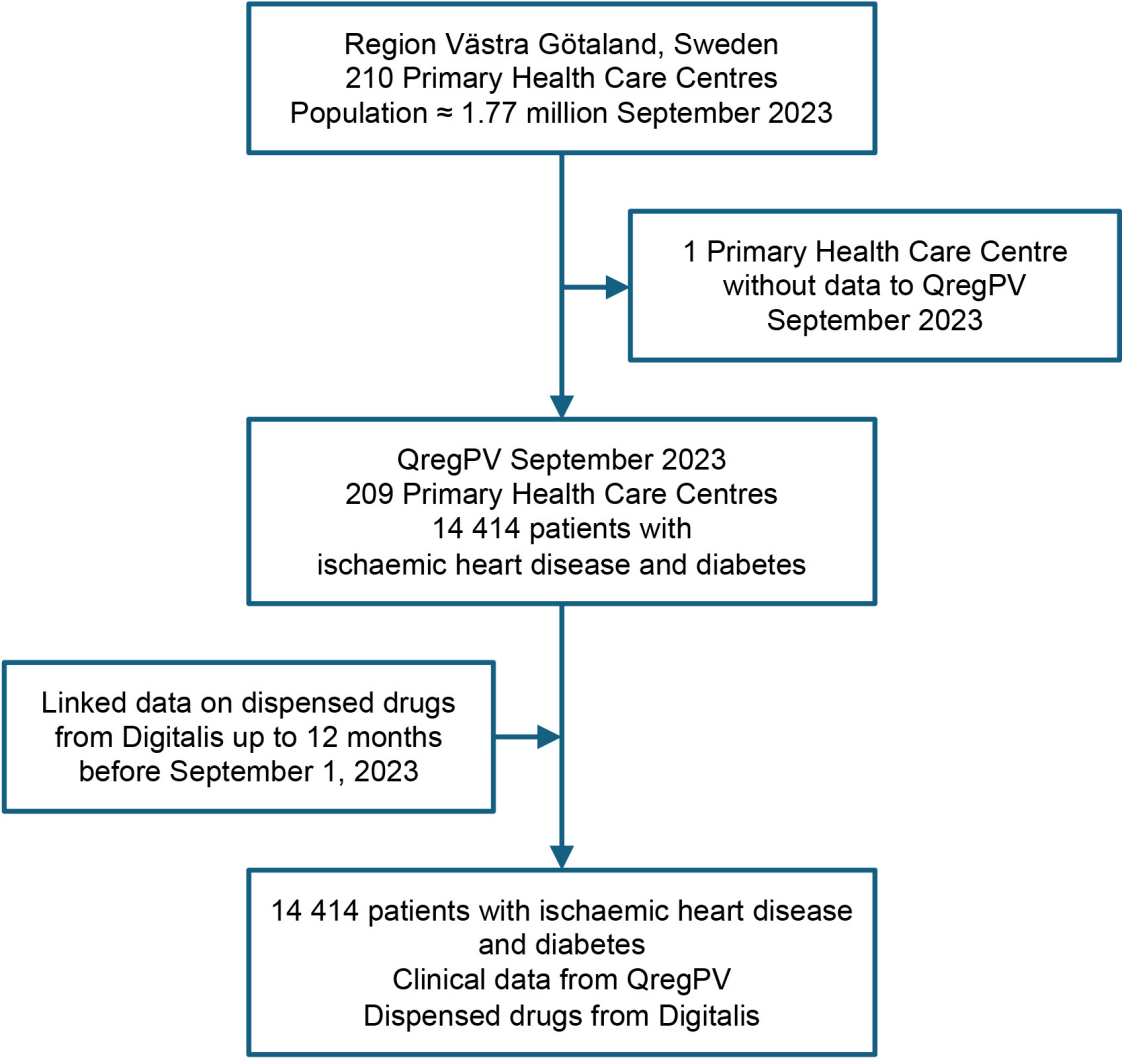
Flowchart illustrating the patients included in the study. QregPV refers to the regional quality of care register for primary care in Region Västra Götaland, Sweden. Digitalis is the regional prescribed drug registry, containing data on all dispensed medications.

The study included 14 414 patients with concomitant IHD and T2D (figure 1). Patient characteristics and dispensed medications are presented in total and by sex in table 1 and by age categories in online supplemental table S3. The mean age was 74.4 years and 30.6% were women. Regarding CV risk factors, 12.7% were smokers, the mean blood pressure was 130/73 mm Hg, the mean glycated haemoglobin (HbA1c) was 52.8 mmol/mol and the mean low-density lipoprotein cholesterol (LDL-C) was 1.86 mmol/L. Numerically, women were older than men, had higher systolic blood pressure, higher HbA1c and higher LDL-C. Diagnosis of hypertension was present among 89.5% in both women and men, while asthma (14.6% vs 7.7%) and COPD (11.3% vs 9.0%) were more common in women. Lipid-lowering therapy was dispensed (up to 120 days prior to the index date) to 82.8% of the patients, less frequently in women (77.2%) than in men (85.3%). Similarly, antithrombotic therapy was dispensed to 86.9% and was less common in women (84.2%) than in men (88.1%).

**Table 1:** Patient characteristics and dispensed drugs

	All patients	Men	Women	Missing, %
Number of patients	14 414	10 004	4410	
Women, n (%)	4410 (30.6)	0 (0.0)	4410 (100.0)	0.0
Age, years (SD)	74.4 (10.0)	73.4 (9.8)	76.6 (10.2)	0.0
Age categories, years, n (%)				0.0
<50	192 (1.3)	150 (1.5)	42 (1.0)	
50–59	1000 (6.9)	771 (7.7)	229 (5.2)	
60–69	3023 (21.0)	2288 (22.9)	735 (16.7)	
70–79	5518 (38.3)	3941 (39.4)	1577 (35.8)	
80–89	3970 (27.5)	2528 (25.3)	1442 (32.7)	
≥90	711 (4.9)	326 (3.3)	385 (8.7)	
Systolic BP, mm Hg (SD)	130.3 (16.4)	129.8 (16.0)	131.4 (17.5)	9.2
Diastolic BP, mm Hg (SD)	73.5 (10.2)	73.7 (10.2)	73.1 (10.3)	9.1
BP<130/80 mm Hg, n (%)	5113 (39.1)	3616 (39.6)	1497 (38.0)	9.2
BP<140/90 mm Hg, n (%)	9503 (72.6)	6790 (74.3)	2713 (68.8)	9.2
HbA1c, mmol/mol (SD)	52.8 (12.5)	52.6 (12.4)	53.3 (12.9)	7.9
HbA1c<52 mmol/mol, n (%)	7389 (55.6)	5206 (56.1)	2183 (54.6)	7.9
Total cholesterol, mmol/L (SD)	3.60 (1.00)	3.46 (0.94)	3.92 (1.06)	12.6
LDL-C, mmol/L (SD)	1.86 (0.86)	1.79 (0.82)	2.02 (0.94)	8.3
LDL-C<1.4 mmol/L, n (%)	3743 (28.3)	2876 (30.9)	867 (22.2)	8.3
LDL-C<1.8 mmol/L, n (%)	7281 (55.1)	5417 (58.2)	1864 (47.7)	8.3
Triglycerides, mmol/L (SD)	1.67 (1.01)	1.64 (1.01)	1.76 (1.00)	36.5
Height, cm (SD)	171.1 (9.7)	175.4 (7.2)	161.1 (6.8)	4.7
Weight, kg (SD)	84.9 (17.4)	88.5 (16.5)	76.1 (16.4)	20.0
Body mass index, kg/m^2^ (SD)	28.9 (5.2)	28.7 (4.9)	29.3 (5.9)	21.0
Waist circumference, cm (SD)	105.1 (13.3)	106.8 (12.8)	100.7 (13.7)	60.4
Smoker, n (%)	1301 (12.7)	948 (13.0)	353 (12.0)	29.1
Comorbidities, n (%)				0.0
Hypertension	12 895 (89.5)	8950 (89.5)	3945 (89.5)	
Asthma	1413 (9.8)	770 (7.7)	643 (14.6)	
COPD	1400 (9.7)	902 (9.0)	498 (11.3)	
Antidiabetic therapy, n (%)				0.0
Metformin	8147 (56.5)	5929 (59.3)	2218 (50.3)	
SGLT2i	5357 (37.2)	4085 (40.8)	1272 (28.8)	
Insulin	3482 (24.2)	2367 (23.7)	1115 (25.3)	
DPP-4 inhibitor	1790 (12.4)	1220 (12.2)	570 (12.9)	
GLP-1 RA	1445 (10.0)	1038 (10.4)	407 (9.2)	
Repaglinide	625 (4.3)	429 (4.3)	196 (4.4)	
Sulfonylurea	329 (2.3)	230 (2.3)	99 (2.2)	
Thiazolidinedione	283 (2.0)	207 (2.1)	76 (1.7)	
SGLT2i and/or GLP-1 RA	6071 (42.1)	4553 (45.5)	1518 (34.4)	
SGLT2i and GLP-1 RA	731 (5.1)	570 (5.7)	161 (3.7)	
Any antidiabetic therapy	11 994 (83.2)	8496 (84.9)	3498 (79.3)	
Lipid-lowering therapy, n (%)				0.0
Statin	11 511 (79.9)	8241 (82.4)	3270 (74.1)	
Ezetimibe	2680 (18.6)	1988 (19.9)	692 (15.7)	
PCSK9-inhibitor	143 (1.0)	95 (0.9)	48 (1.1)	
Other lipid-lowering therapy	115 (0.8)	78 (0.8)	37 (0.8)	
Any lipid-lowering therapy	11 935 (82.8)	8529 (85.3)	3406 (77.2)	
Antithrombotic therapy, n (%)				0.0
Acetylsalicylic acid	8344 (57.9)	5883 (58.8)	2461 (55.8)	
NOAC	3247 (22.5)	2286 (22.9)	961 (21.8)	
P2Y_12_ inhibitor	1605 (11.1)	1129 (11.3)	476 (10.8)	
Warfarin	369 (2.6)	284 (2.8)	85 (1.9)	
Low molecular weight heparin	105 (0.7)	69 (0.7)	36 (0.8)	
Any anti-thrombotic therapy	12 530 (86.9)	8817 (88.1)	3713 (84.2)	
Cardiovascular drugs, n (%)				0.0
Beta-blocker	10 313 (71.5)	7087 (70.8)	3226 (73.2)	
Angiotensin-receptor blocker	5467 (37.9)	3687 (36.9)	1780 (40.4)	
Calcium-channel blocker	5071 (35.2)	3441 (34.4)	1630 (37.0)	
ACE inhibitor	4703 (32.6)	3505 (35.0)	1198 (27.2)	
Loop diuretic	3035 (21.1)	1864 (18.6)	1171 (26.6)	
Thiazide diuretic	2189 (15.2)	1472 (14.7)	717 (16.3)	
Mineral receptor antagonist	1874 (13.0)	1325 (13.2)	549 (12.4)	
Isosorbide mononitrate	2193 (15.2)	1324 (13.2)	869 (19.7)	
Glyceryl nitrate	1799 (12.5)	1145 (11.4)	654 (14.8)	
ARNI	374 (2.6)	322 (3.2)	52 (1.2)	
Other antihypertensive	361 (2.5)	265 (2.6)	96 (2.2)	

Numbers are presented as mean (SD) for continuous variables, and as n (%) for categorical variables.

Drugs were dispensed within 120 days prior to 1 September 2023.

ARNI, angiotensin receptor neprilysin inhibitor; BP, blood pressure; COPD, chronic obstructive pulmonary disease; DPP-4, dipeptidyl peptidase-4; GLP-1 RA, glucagon-like peptide 1 receptor agonists; HbA1c, glycated haemoglobin; LDL-C, low-density lipoprotein cholesterol; NOAC, non-vitamin K antagonist oral anticoagulant; PCSK9, proprotein convertase subtilisin/kexin type 9; SGLT2i, sodium–glucose cotransporter 2 inhibitor.

### Antidiabetic therapy

Any type of antidiabetic therapy was dispensed to 79.3% in women and 84.9% in men (table 1). All classes of dispensed antidiabetic medications were predominantly prescribed from PHCCs, with only a minority prescribed from secondary care (online supplemental figure S1). Metformin was the most common medication, dispensed to over half of the patients. The second most dispensed medication was SGLT2i (37.2%), dispensed less often to women than to men (28.8% vs 40.8%; age-adjusted OR 0.64 (95% CI 0.59 to 0.70)). Dispensed GLP-1 RA was less common (10.0%) and was similarly dispensed to women and men (9.2% vs 10.4%, age-adjusted OR 1.04 (95% CI 0.92 to 1.18)). An SGLT2i and/or a GLP-1 RA was dispensed to 42.1%, whereas a combination of SGLT2i and GLP-1 RA was dispensed to 5.1%.

**Figure 2:**
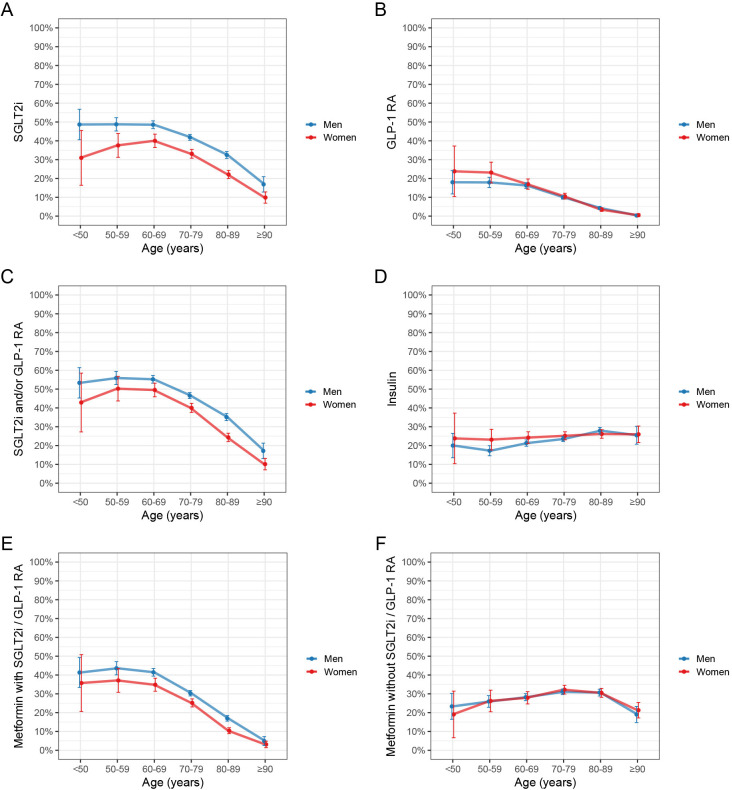
Proportions of patients with dispensed drugs according to age and sex. (A) SGLT2i, (B) GLP-1 RA, (C) SGLT2i and/or GLP-1 RA, (D) insulin, (E) metformin with SGLT2i and/or GLP-1 RA and (F) metformin without SGLT2i or GLP-1 RA. Drugs were dispensed within 120 days prior to 1 September 2023. The error bars represent 95% CIs. GLP-1 RA, glucagon-like peptide 1 receptor agonists; SGLT2i, sodium-glucose cotransporter 2 inhibitors.

Figure 2 presents dispensed SGLT2i, GLP-1 RA, insulin and metformin up to 120 days prior to the index date according to sex and age. Overall, the highest dispensation rates of SGLT2i and GLP-1 RA were seen among younger patients and declined with age. The highest dispensation rate for SGLT2i was seen among 60- to 69-year-old patients (46.5%) and was lowest among those 90 years and older (13.1%), p<0.001 for trend in age. GLP-1 RA was most common among <50-year-olds (19.3%), whereas dispensed to only 0.4% of those 90 years and older, p<0.001 for trend in age. In contrast, this pattern was not observed for insulin or metformin without SGLT2i or GLP-1 RA, which were dispensed to 24.2% and 29.5% of patients, respectively. Sensitivity analyses of dispensed medications up to 1 year prior to the index date showed similar dispensation patterns, although with higher absolute numbers (SGLT2i 41.8% and GLP-1 RA 12.3%; online supplemental figure S2).

### Variation in dispensed SGLT2i and GLP-1 RA across primary healthcare centres

**Figure 3:**
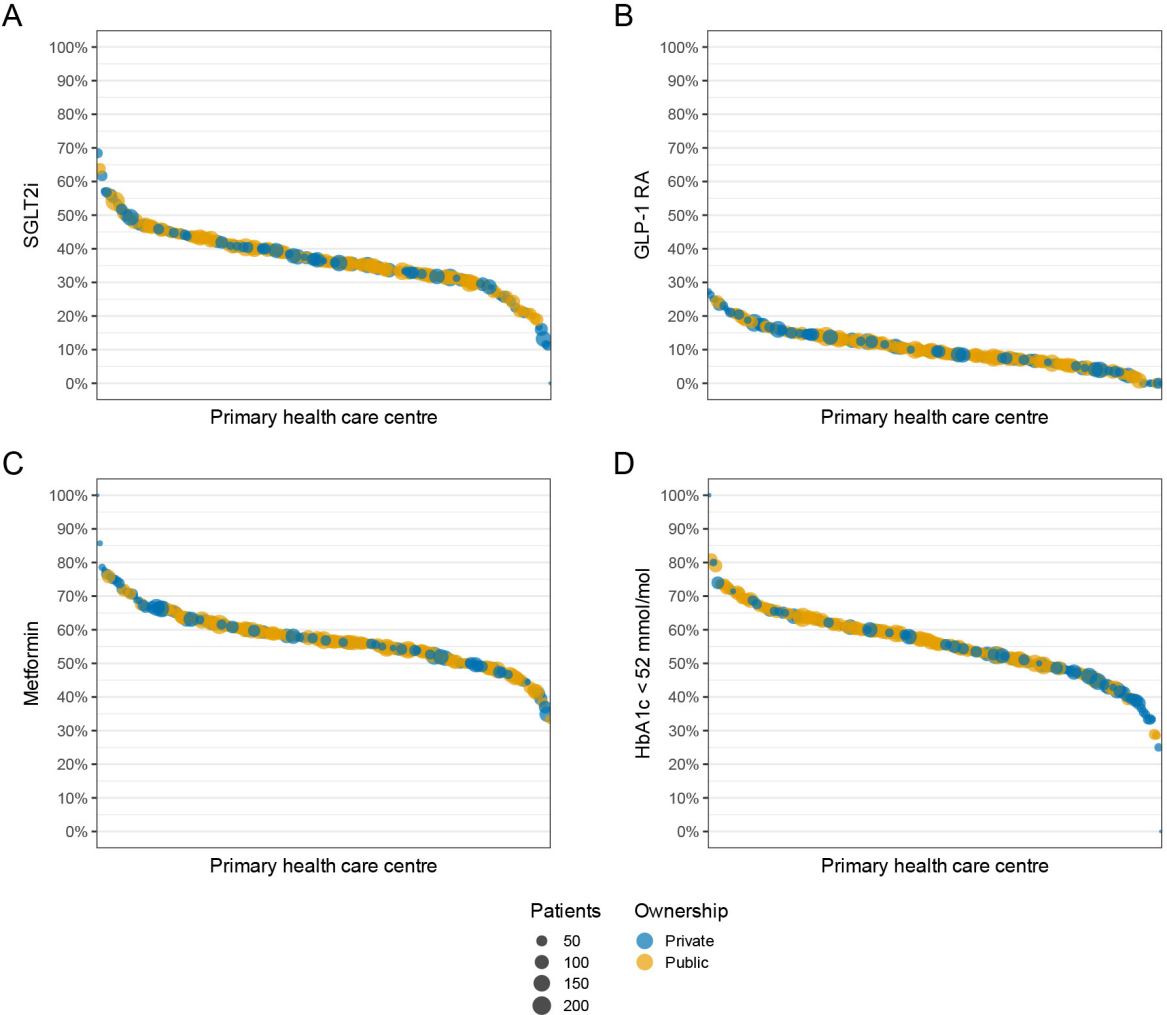
Variation in dispensed drugs and glycaemic control across different primary healthcare centres. The panel figure shows the proportion of patients with (A) SGLT2i, (B) GLP-1 RA, (C) metformin and (D) HbA1c<52 mmol/mol. Drugs were dispensed within 120 days prior to 1 September 2023. Each dot represents a unique primary healthcare centre. The size of the dots corresponds to the number of patients at the different primary healthcare centres and the colour corresponds to whether public or private ownership. GLP-1 RA, glucagon-like peptide 1 receptor agonists; HbA1c, glycated haemoglobin; SGLT2i, sodium-glucose cotransporter 2 inhibitors.

The proportions of patients with dispensed SGLT2i and GLP-1 RA varied across PHCCs. The median SGLT2i dispensation rate was 36.4%, with a 2.5th–97.5th percentile range of 17.1%–56.4% (figure 3A). Correspondingly, the median GLP-1 RA dispensation rate was 9.5%, with a 2.5th–97.5th percentile range of 0.0%–23.4% (figure 3B). For comparison, dispensation rates of metformin also varied across PHCCs but were generally higher, with a 2.5th–97.5th percentile range of 41.1%–75.8% (figure 3C). The median proportion of patients with HbA1c<52 mmol/mol was 56.0%, with a 2.5th–97.5th percentile range of 33.3%–73.5% across PHCCs (figure 3D).

**Figure 4:**
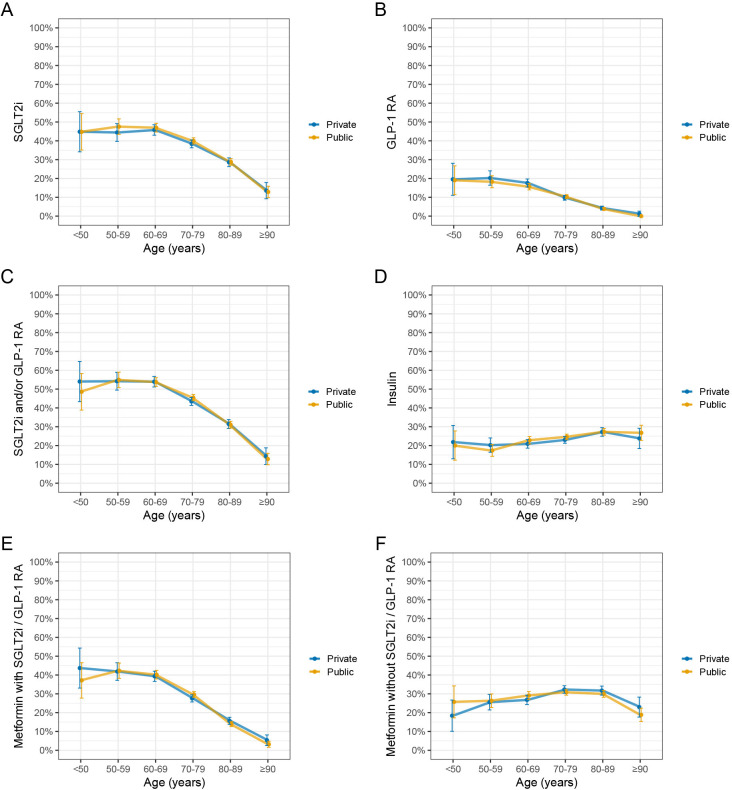
Proportions of patients with dispensed drugs according to age and primary healthcare centre ownership. (A) SGLT2i, (B) GLP-1 RA, (C) SGLT2i and/or GLP-1 RA, (D) insulin, (E) metformin with SGLT2i and/or GLP-1 RA and (F) metformin without SGLT2i or GLP-1 RA. Drugs were dispensed within 120 days prior to 1 September 2023. The error bars represent 95% CIs. GLP-1 RA, glucagon-like peptide 1 receptor agonists; SGLT2i, sodium-glucose cotransporter 2 inhibitors.

Adjusted for sex and age, there were no significant differences among patients at privately versus publicly owned PHCCs regarding dispensation of SGLT2i (sex and age adjusted OR 0.95 (95% CI 0.85 to 1.06)), GLP-1 RA (sex and age adjusted OR 1.06 (95% CI 0.89 to 1.26)) or metformin (sex and age adjusted OR 1.00 (95% CI 0.91 to 1.11)). However, the odds of patients having HbA1c<52 mmol/mol were lower among private versus publicly owned PHCCs (sex and age adjusted OR 0.79 (95% CI 0.71 to 0.88)). Figure 4 presents dispensed SGLT2i, GLP-1 RA, insulin and metformin according to age and PHCC ownership. Throughout, the overlapping 95% CIs indicate no differences between privately and publicly owned PHCCs. Similar patterns with overlapping CIs for privately and publicly owned PHCCs were seen for dispensed drugs up to 1 year prior to the index date (online supplemental figure S3).

The results of multilevel regression models to estimate variability in dispensation rates of SGLT2i and GLP-1 RA expressed as MOR across PHCCs are presented in table 2. In model 3, adjusting for patient and PHCC characteristics, the MOR between different PHCCs regarding dispensation of SGLT2i was 1.29 (95% CI 1.23 to 1.36). The corresponding MOR for dispensation of GLP-1 RA was 1.48 (95% CI 1.37 to 1.62). Sensitivity analyses using splines for continuous variables showed nearly identical results (online supplemental table S4). Consistent with results from ordinary logistic regression models, the fully adjusted multilevel regression model 3 showed no differences in the dispensation of SGLT2i between privately and publicly owned PHCCs (OR 0.97 (95% CI 0.87 to 1.08)). Similarly, no difference was observed for the dispensation of GLP-1 RA (OR 1.07 (95% CI 0.90 to 1.26)). Direct standardised rates of SGLT2i and GLP-1 RA usage across PHCCs are plotted in online supplemental figure S4.

**Table 2:** Variation across primary healthcare centres regarding dispensed SGLT2i and GLP-1 RA

	Model 1		Model 2		Model 3	
	MOR	95% CI	MOR	95% CI	MOR	95% CI
SGLT2 inhibitor	1.29	1.23 to 1.36	1.29	1.23 to 1.36	1.29	1.23 to 1.36
GLP-1 RA	1.46	1.35 to 1.59	1.51	1.40 to 1.65	1.48	1.37 to 1.62

Patients with ischaemic heart disease and diabetes in Region Västra Götaland, Sweden. Multilevel regression models were used to estimate MOR with 95% CIs. Model 1: unadjusted. Model 2: adjusted for patient characteristics: age, sex and comorbidities: hypertension, asthma and chronic obstructive pulmonary disease. Model 3: adjusted for patient characteristics: age, sex, comorbidities and primary healthcare centre characteristics: care need index, adjusted clinical groups, number of enrolled patients and public/private ownership. Dispensed medications were registered up to 120 days before the index date, 1 September 2023.

GLP-1 RA, glucagon-like-peptide 1 receptor agonists; MOR, median OR; SGLT2, sodium–glucose cotransporter 2.

### Potential additional cost of SGLT2i and GLP-1 RA

In 2023, the annual pharmacy cost for an SGLT2i (empagliflozin 10 mg or dapagliflozin 10 mg daily) was €471 (5121 SEK), compared with €1246 (13 548 SEK) for a GLP-1 RA (semaglutide 0.25–1 mg or dulaglutide 0.75–4 mg weekly). The cost refers to the combined payment by patients and the reimbursement system. Of the estimated 14 414 patients in VGR with IHD and T2D, 42.1% had already been dispensed an SGLT2i and/or GLP-1 RA in 2023, leaving 57.9% (8345 patients) untreated. If those patients were dispensed an SGLT2i or a GLP-1 RA according to current ESC guidelines, the additional annual cost would be €3.9 million (43 million SEK) or €10.4 million (113 million SEK), respectively.

## Discussion

In this large contemporary (2023) Swedish cross-sectional study of 14 414 primary care patients with coexisting IHD and T2D, treatment with SGLT2i and GLP-1 RA was underused relative to current ESC guidelines, which generally recommend at least one of these treatments. SGLT2i were dispensed to 37.2% of patients, but dispensation was disproportionate across age and sex, with higher usage among younger patients and men. GLP-1 RA was dispensed to 10.0% of patients, also more frequently among younger patients, but without any difference across sexes. Overall, 42.1% of patients received either an SGLT2i, a GLP-1 RA or both. Dispensation rates varied substantially across different PHCCs, but no significant differences were observed between privately and publicly owned centres. If all untreated patients were dispensed either an SGLT2i or a GLP-1 RA, the additional annual cost would be considerable, €3.9 million (43 million SEK) and €10.4 million (113 million SEK), respectively.

### Comparison to previous studies

To the best of our knowledge, this study is the first to include virtually all patients with concomitant IHD and T2D in primary care in a large region to explore variation across PHCCs regarding dispensed SGLT-2i and GLP-1 RA. From the USA, variation between different Veterans Affairs primary care facilities (approximately 98% men) in prescriptions of SGLT2i and GLP-1 RA has been reported in a large study with data from 2020, including patients with established atherosclerotic cardiovascular disease (ASCVD) and T2D.^5^ Compared with our study, the overall rates of SGLT2i (11.2% vs 37.2%) and GLP-1 RA (8.0% vs 10.0%) were lower in the US study. In addition, the facility-level variations between different healthcare centres expressed as adjusted median rate/ORs were higher in the US study than in ours regarding both the use of SGLT2i (1.55 (95% CI 1.46 to 1.63) vs 1.29 (95% CI 1.23 to 1.36)) and GLP-1 RA (1.78 (95% CI 1.65 to 1.90) vs 1.48 (95% CI 1.37 to 1.62)). Other studies from the US Veteran Affairs system have noted similar facility-level variation in SGLT2i prescriptions among patients with ASCVD, T2D and chronic kidney disease, as well as among those with ASCVD, T2D and heart failure.^6 7 18^ In the global observational DISCOVER study, which included patients with T2D from countries with various socioeconomic backgrounds, the variability between countries in the use of SGLT2i and/or GLP-1 RA was even higher (MOR 3.48).^19^

In our study, 42.1% of patients with IHD and T2D were dispensed an SGLT2i, a GLP-1 RA or both—higher than in earlier primary care studies. The use of SGLT2i and GLP-1 RA in our study can be viewed in relation to the general T2D population in Sweden, where, according to the Swedish National Diabetes Register, 19% of patients were prescribed an SGLT2i and 13% a GLP-1 RA in 2023.^20^ In a Portuguese study using 2019–2020 data, 36.1% of patients with T2D and ASCVD were prescribed either drug.^21^ Lower rates were reported in Belgium, though prescriptions increased from 2019 to 2023 (SGLT2i: 1.5%–9.6%; GLP-1 RA: ≈4%–11.9%).^22^ In the Netherlands, using 2022 data, only 10.3% of patients with T2D and ischaemic CVD, chronic kidney disease or heart failure received an SGLT2i, and 70% of those not treated had no contraindication.^23^ In Canada, based on 2018–2020 data, 19.4% of patients with T2D and an additional cardiorenal indication were prescribed SGLT2i or GLP-1 RA, with minimal difference compared with those without an additional cardiorenal indication.^24^ As in our study, younger age and male sex were associated with higher prescription rates.

Temporal trends of increased use of either SGLT2i or GLP-1 RA in patients with T2D and ASCVD have been reported in large US real-world studies based on a mix of inpatient and outpatient electronic health records.^25 26^ Similar to our study, higher use was observed among younger patients, and one study also noted lower use of SGLT2i in women than men.^25^ A Korean hospital-based study with 2019–2020 data likewise found lower use of SGLT2i in women after a CVD event.^27^ Data from SEPHIA, the Swedish secondary prevention register within the national SWEDEHEART register, show rising use of SGLT2i/GLP-1 RA among patients with T2D after acute myocardial infarction (43% in 2021, 60% in 2022, 65% in 2023).^3^ These patients are later referred to primary care for follow-up and prescription renewal, contributing to increased use. However, the SEPHIA population differs from ours, as it excludes patients over 80 and underrepresents those with multimorbidity or frailty, which has been associated with lower initiation rates of SGLT2i and GLP-1 RA.^28^

### Clinical implications

A meta-analysis of randomised placebo-controlled trials (RCTs) of SGLT2i showed an 11% relative risk reduction in the composite outcome of myocardial infarction, stroke and CV death.^29^ Similarly, a meta-analysis of RCTs of GLP-1 RA in patients with T2D and established or high risk of ASCVD reported a 14% relative risk reduction in the same composite outcome, corresponding to a number needed to treat of 65 over 3 years of follow-up.^30^ As both SGLT2i and GLP-1 RA have demonstrated cardioprotective benefits in patients with T2D and ASCVD, increased use of these agents would be clinically advantageous. Considering the wide variation in use across PHCCs, which likely reflects inequity in care delivery, efforts to increase uptake are particularly warranted at PHCCs with the lowest usage. Strategies to increase the use of SGLT2i and GLP-1 RA could include targeted feedback to PHCCs with low use, educational efforts aimed at prescribers to increase awareness of regional guidelines, and qualitative studies to interview prescribers as well as patients about awareness and attitudes concerning medical cardioprotective treatment. For example, significantly increased prescription rates of SGLT2i and GLP-1 RA were recently reported from a US Veteran Affairs multidisciplinary quality improvement intervention.^31^

Our study demonstrates a sex disparity, with women being dispensed SGLT2i to a lesser extent than men. Higher susceptibility to urinary tract infections among women, with potential subsequent discontinuation of SGLT2i, may possibly be reflected in the lower use of SGLT2i observed in women in our study. As current guidelines are sex-neutral, increased use of SGLT2i among women appears warranted. Previous studies have similarly reported lower prescription rates of CV medications in women compared with men in primary care.^2^ In line with this, we have recently shown that women with IHD have poorer risk factor control and lower use of antithrombotic and lipid-lowering therapy than men, with substantial variability in risk factor control across PHCCs.^32^

The comparatively high cost of SGLT2i relative to other glucose-lowering medications has been identified as a barrier to wider clinical adoption.^33^ This economic barrier is likely to vary between countries depending on national reimbursement systems and insurance coverage. It may also vary within countries; for example, in Sweden, where the cost of medications prescribed at PHCCs in different counties may be covered either by the individual PHCC or at the county level. As GLP-1 RA are significantly more expensive than SGLT2i, the financial obstacle to prescribing them is likely even greater. In Sweden, patients’ out-of-pocket expenses for outpatient pharmaceuticals are relatively low; in 2023, the annual cost was capped at 2600 SEK (approximately €240), with the remaining cost subsidised through public funding.^34^ In VGR, this cost is absorbed by the individual PHCCs, which are publicly funded regardless of public or private ownership. Consequently, when many patients at a PHCC are prescribed high-cost medications, the financial burden primarily falls on the centre itself.

We estimated that the additional annual cost in VGR 2023 would be €3.9 million (43 million SEK) or €10.4 million (113 million SEK) if all untreated patients with IHD and T2D were dispensed an SGLT2i or a GLP-1 RA, respectively, in accordance with ESC guidelines. Extrapolated to the Swedish national level (population 10.6 million), the corresponding additional costs in 2023 are estimated at €23.5 million (256 million SEK) or €62.3 million (677 million SEK), respectively. In VGR, the cost of an SGLT2i to all untreated patients with IHD and T2D would represent a maximum of 3.2% of the total €122.1 million (1321 million SEK) 2023 prescription costs at PHCCs,^35^ while a GLP-1 RA would account for a maximum of 8.6%.

Although such costs may be justified from a broader health economic perspective, they can be substantial at the individual PHCC level, where daily horizontal prioritisation between patient groups poses clinical and financial challenges. In different national healthcare systems, however, the additional cost burden may fall on different levels: the individual patient, health insurance or the central government. Some patients are also unsuitable for these therapies due to contraindications or adverse effects. Applying a more conservative target—treating at least 70% of patients with IHD and T2D up to 75 years of age, as recommended by the Swedish Society for Diabetology at the time—would reduce projected costs.^36^

### Strengths and limitations

Our study has several strengths. First, it explores contemporary (2023) use of SGLT2i and GLP-1 RA among patients with IHD and T2D in 99.5% of PHCCs in a region of 1.8 million inhabitants. This near-complete coverage minimises selection bias and ensures high internal validity. It also reflects the heterogeneity of primary care, encompassing both patients with recent acute coronary events and those diagnosed with IHD decades earlier. However, eligible patients without a recorded diagnosis of T2D or IHD are not reported to QregPV. We consider the risk of resulting selection bias to be small given the relatively large number of patients included in the study. Second, we use complete individual-level data on dispensed medications, which more accurately reflect patients’ actual use compared with prescribed medication data. However, dispensation data are less directly linked to individual PHCCs than prescription data, which may limit the ability to attribute prescribing patterns to specific centres. Third, by using data on PHCC-level, we can report facility variations in use of SGLT2i and GLP-1 RA not previously reported. The study also has some limitations. Although we have PHCC-level data on CNI and ACG, we did not have access to individual socioeconomic data, which have been associated with use of SGLT2i and GLP-1 RA.^2437^,^39^ Possibly, such socioeconomic factors could explain some of the unexplained PHCC-level variation in dispensation rates in our study. Furthermore, in the calculation of the median MOR to reflect inter-practice variation, we did not have access to individual healthcare provider continuity or characteristics, such as age, sex and level of experience, which may influence prescribing patterns. Given the study design, we could not distinguish inappropriate non-use from clinically justified non-prescription, such as intolerance and patient preference. We had no data on frailty or contraindications to SGLT2i or GLP-1 RA, which may partly affect the estimation of their use. Also, we had no data on kidney function and limited data on comorbidities, for example, heart failure and chronic kidney disease, which are also indications for SGLT2i. Interestingly, though, lower use of SGLT2i and GLP-1 RA has been reported among patients with T2D and other cardiorenal indications than without.^40^ We assumed that patients with diabetes in QregPV have T2D, as patients with type 1 diabetes are not primarily managed in primary care. However, we cannot rule out that a small proportion of patients with type 1 diabetes may have been included in our study. We do not believe that this has had any significant impact on our results. Our use of dispensation records within 120 days before the index date may have overestimated treatment use, as some patients may have discontinued the medication after dispensation. Underestimation is also possible. However, we believe that the 1-year dispensation rates, which were slightly higher, indicate a reasonable upper bound for treatment use. During the study period, there was a shortage of subcutaneously administered GLP-1 RA across the European Union, which may have affected the prescription and dispensation rates.^41^ Furthermore, variability in drug pricing and reimbursement policies across and within countries may affect the generalisability of our results to other healthcare settings.

## Conclusions

This large real-world study of patients with IHD and concomitant T2D in Swedish primary care shows higher usage of SGLT2i and GLP-1 RA than most previous reports. However, both medication classes remain underutilised relative to ESC guidelines, particularly among older patients and, in the case of SGLT2i, also among women. The study further reveals substantial variation in use across PHCCs, but with no difference between public and private providers. These findings highlight the need to reduce unwarranted variation in prescribing practices and to address the implementation challenges associated with costly secondary prevention cardioprotective therapies.

## Supplementary material

10.1136/bmjopen-2025-110395 online supplemental file 1

## Data Availability

Data are available upon reasonable request.
