# Supplementary material for: Use of SGLT2 inhibitors and GLP-1 receptor agonists in patients with ischaemic heart disease and type 2 diabetes in Swedish primary care: a cross-sectional analysis of regional primary care registry data (QregPV)
Source: BMJ Open. 2026 Feb 2;16(2):e110395. doi: 10.1136/bmjopen-2025-110395 (PMC12878446; doi:10.1136/bmjopen-2025-110395)
Supplement: online supplemental file 1 [file bmjopen-16-2-s001.pdf]

Supplemental Material

This supplemental material has been provided by the authors to give readers additional information about their work.

Supplement to:

Use of SGLT2-inhibitors and GLP-1 receptor agonists in patients with ischaemic heart disease and type 2 diabetes in Swedish primary care: a cross-sectional analysis of regional primary care registry data (QregPV)

Tobias Andersson, Johan-Emil Bager, Margareta Hellgren, Maria Åberg,
Georgios Mourtzinis

Supplementary Table S1. Variables in QregPV. All primary health care centres in Region Västra Götaland report monthly individual data to QregPV which is updated monthly.

**Variable**	**Time of registration**
Age (years)	Monthly
Sex (man, woman)	
Systolic blood pressure (mmHg)	Last registration within 450 days
Diastolic blood pressure (mmHg)	Last registration within 450 days
HbA1c (mmol/mol)	Last registration within 450 days
Total cholesterol (mmol/L)	Last registration within 900 days
LDL-cholesterol (mmol/L)	Last registration within 900 days
Triglycerides (mmol/L)	Last registration within 900 days
Height (cm)	Last registration without time limit
Weight (kg)	Last registration within 450 days
Waist circumference (cm)	Last registration within 450 days
Smoking (yes, no)	Last registration within 450 days
Hypertension, ICD-10: I10–I15	Within 900 days
Ischemic heart disease, ICD-10: I20–I25	Within 900 days
Diabetes mellitus, ICD-10: E10–E14	Within 900 days
Asthma, ICD-10: J45	Within 900 days
COPD, ICD-10: J44	Within 900 days
Enlisted primary health care centre	Monthly

LDL: low-density lipoprotein. ICD: International classification of diseases. COPD: chronic obstructive pulmonary disease.

Supplementary Table S2. Anatomical therapeutical chemical (ATC) classification system codes for dispensed drugs in the study.

	**ATC-code**
**Antidiabetic therapy**	
Metformin	A10BA02<br>A10BD05<br>A10BD07<br>A10BD08<br>A10BD10<br>A10BD11<br>A10BD13<br>A10BD15<br>A10BD16<br>A10BD20<br>A10BD23
SGLT2i	A10BK<br>A10BD15<br>A10BD16<br>A10BD19<br>A10BD20<br>A10BD21<br>A10BD23<br>A10BD24
Insulin	A10A
DPP-4 inhibitor	A10BH<br>A10BD07<br>A10BD08<br>A10BD09<br>A10BD10<br>A10BD11<br>A10BD13<br>A10BD19<br>A10BD21<br>A10BD24
GLP-1 RA	A10BJ
Repaglinide	A10BX02
Sulfonylurea	A10BB<br>A10BD06
Thiazolidinedione	A10BG<br>A10BD05<br>A10BD06<br>A10BD09
**Lipid-lowering therapy**	
Statin	C10AA<br>C10BA
Ezetimibe	C10AX09<br>C10BA
PCSK9-inhibitor	C10AX13<br>C10AX14
Other lipid-lowering therapy	C10AB<br>C10AC<br>C10AX06

**Antithrombotic therapy**	
Acetylsalicylic acid	B01AC06
NOAC	B01AF<br>B01AE07
$P2Y_{12}$ inhibitor	B01AC04<br>B01AC24<br>B01AC22
Warfarin	B01AA03
Low molecular weight heparin	B01AB
**Cardiovascular drugs**	
Beta-blocker	C07
Angiotensin-receptor blocker	C09C<br>C09D
Calcium-channel blocker	C08<br>C09BB<br>C09DB<br>C07FB
ACE inhibitor	C09A<br>C09B
Loop diuretic	C03C
Thiazide diuretic	C03A<br>C03B<br>C03EA<br>C09BA<br>C09DA
Mineral receptor antagonist	C03DA
Isosorbide mononitrate	C01DA14
Glyceryl nitrate	C01DA02
ARNI	C09DX04
Other antihypertensive	C02A<br>C02CA<br>C02D<br>C09XA

SGLT2i: sodium–glucose cotransporter 2 inhibitor. DPP-4: dipeptidyl peptidase-4. GLP-1 RA: glucagon-like peptide 1 receptor agonists. PCSK9: proprotein convertase subtilisin/kexin type 9. NOAC: non-vitamin K antagonist oral anticoagulant. ACE: angiotensin converting enzyme. ARNI: angiotensin receptor neprilysin inhibitor.

Supplementary Table S3. Patient characteristics, and dispensed drugs up to 120 days before the index date, according to age categories.

	**All patients**	**<50 years**	**50-59 years**	**60-69 years**	**70-79 years**	**80-89 years**	**≥90 years**	**Missing, %**
Number of patients	14 414	192	1000	3023	5518	3970	711	
Women, n (%)	4410 (30.6)	42 (21.9)	229 (22.9)	735 (24.3)	1577 (28.6)	1442 (36.3)	385 (54.1)	0.0
Age, years (SD)	74.4 (10.0)	45.3 (4.3)	55.7 (2.8)	65.1 (2.8)	74.8 (2.8)	83.7 (2.7)	92.2 (2.2)	0.0
Systolic BP, mmHg (SD)	130.3 (16.4)	125.8 (15.7)	128.3 (15.9)	129.2 (15.5)	130.2 (16.0)	131.5 (17.1)	133.0 (19.8)	9.2
Diastolic BP, mmHg (SD)	73.5 (10.2)	80.3 (10.8)	78.5 (9.9)	76.2 (9.9)	73.2 (9.7)	71.1 (10.2)	70.3 (10.0)	9.1
BP <130/80 mmHg, n (%)	5113 (39.1)	59 (36.2)	302 (34.1)	1090 (39.8)	2021 (39.7)	1409 (39.2)	232 (37.8)	9.2
BP <140/90 mmHg, n (%)	9503 (72.6)	120 (73.6)	668 (75.5)	2096 (76.6)	3756 (73.8)	2480 (68.9)	383 (62.5)	9.2
HbA1c, mmol/mol (SD)	52.8 (12.5)	53.6 (16.0)	53.6 (14.0)	52.7 (13.0)	52.0 (11.7)	53.6 (12.5)	54.9 (12.9)	7.9
HbA1c <52 mmol/mol, n (%)	7389 (55.6)	95 (58.6)	491 (54.4)	1602 (57.8)	3003 (58.2)	1904 (52.0)	294 (47.3)	7.9
Total cholesterol, mmol/L (SD)	3.60 (1.00)	3.61 (1.18)	3.53 (1.10)	3.53 (0.99)	3.54 (0.95)	3.70 (1.01)	4.04 (1.12)	12.6
LDL-C, mmol/L (SD)	1.86 (0.86)	1.92 (1.05)	1.83 (0.91)	1.80 (0.84)	1.80 (0.82)	1.95 (0.87)	2.28 (1.00)	8.3
LDL-C <1.4 mmol/L, n (%)	3743 (28.3)	56 (32.9)	304 (32.3)	897 (31.4)	1596 (30.4)	825 (23.6)	65 (12.9)	8.3
LDL-C <1.8 mmol/L, n (%)	7281 (55.1)	97 (57.1)	555 (59.0)	1660 (58.0)	3023 (57.6)	1756 (50.3)	190 (37.8)	8.3
Triglycerides, mmol/L (SD)	1.67 (1.01)	2.17 (1.54)	1.96 (1.30)	1.79 (1.16)	1.65 (0.93)	1.55 (0.85)	1.53 (0.94)	36.5
Height, cm (SD)	171.1 (9.7)	172.5 (10.3)	173.2 (9.3)	172.4 (9.4)	171.9 (9.5)	169.3 (9.5)	165.3 (9.9)	4.7
Weight, kg (SD)	84.9 (17.4)	98.2 (24.4)	92.9 (18.6)	89.6 (17.8)	85.5 (16.4)	79.2 (15.2)	71.2 (13.9)	20.0
Body mass index, kg/m^2 (SD)	28.9 (5.2)	33.0 (7.3)	31.0 (5.6)	30.1 (5.3)	28.9 (5.1)	27.5 (4.7)	26.0 (4.4)	21.0
Waist circumference, cm (SD)	105.1 (13.3)	111.6 (16.3)	107.5 (13.6)	107.0 (13.1)	105.7 (13.4)	102.1 (12.3)	96.6 (11.3)	60.4
Smoker, n (%)	1301 (12.7)	36 (28.1)	212 (29.1)	414 (18.5)	507 (12.3)	127 (4.7)	5 (1.6)	29.1
Comorbidities, n (%)								0.0
Hypertension	12 895 (89.5)	127 (66.1)	826 (82.6)	2653 (87.8)	5016 (90.9)	3640 (91.7)	633 (89.0)	
Asthma	1413 (9.8)	15 (7.8)	107 (10.7)	315 (10.4)	547 (9.9)	367 (9.2)	62 (8.7)	
COPD	1400 (9.7)	4 (2.1)	45 (4.5)	237 (7.8)	630 (11.4)	431 (10.9)	53 (7.5)	

Antidiabetic therapy, n (%)								0.0
Metformin	8147 (56.5)	120 (62.5)	681 (68.1)	2055 (68.0)	3325 (60.3)	1794 (45.2)	172 (24.2)	
SGLT2i	5357 (37.2)	86 (44.8)	462 (46.2)	1405 (46.5)	2171 (39.3)	1140 (28.7)	93 (13.1)	
Insulin	3482 (24.2)	40 (20.8)	186 (18.6)	666 (22.0)	1324 (24.0)	1083 (27.3)	183 (25.7)	
DPP-4 inhibitor	1790 (12.4)	9 (4.7)	74 (7.4)	275 (9.1)	670 (12.1)	635 (16.0)	127 (17.9)	
GLP-1 RA	1445 (10.0)	37 (19.3)	191 (19.1)	497 (16.4)	559 (10.1)	158 (4.0)	3 (0.4)	
Repaglinide	625 (4.3)	4 (2.1)	41 (4.1)	121 (4.0)	266 (4.8)	170 (4.3)	23 (3.2)	
Sulfonylurea	329 (2.3)	3 (1.6)	31 (3.1)	82 (2.7)	145 (2.6)	60 (1.5)	8 (1.1)	
Thiazolidinedione	283 (2.0)	4 (2.1)	28 (2.8)	95 (3.1)	117 (2.1)	34 (0.9)	5 (0.7)	
SGLT2i and/or GLP-1 RA	6071 (42.1)	98 (51.0)	546 (54.6)	1627 (53.8)	2466 (44.7)	1239 (31.2)	95 (13.4)	
SGLT2i and GLP-1 RA	731 (5.1)	25 (13.0)	107 (10.7)	275 (9.1)	264 (4.8)	59 (1.5)	1 (0.1)	
Any antidiabetic therapy	11 994 (83.2)	153 (79.7)	864 (86.4)	2653 (87.8)	4751 (86.1)	3131 (78.9)	442 (62.2)	
Lipid-lowering therapy, n (%)								0.0
Statin	11 511 (79.9)	139 (72.4)	810 (81.0)	2511 (83.1)	4573 (82.9)	3084 (77.7)	394 (55.4)	
Ezetimibe	2680 (18.6)	60 (31.2)	294 (29.4)	779 (25.8)	1117 (20.2)	416 (10.5)	14 (2.0)	
PCSK9-inhibitor	143 (1.0)	4 (2.1)	19 (1.9)	43 (1.4)	67 (1.2)	10 (0.3)	0 (0.0)	
Other lipid-lowering therapy	115 (0.8)	7 (3.6)	11 (1.1)	37 (1.2)	42 (0.8)	17 (0.4)	1 (0.1)	
Any lipid-lowering therapy	11 935 (82.8)	150 (78.1)	848 (84.8)	2596 (85.9)	4751 (86.1)	3189 (80.3)	401 (56.4)	
Antithrombotic therapy, n (%)								0.0
Acetylsalicylic acid	8344 (57.9)	127 (66.1)	727 (72.7)	2098 (69.4)	3291 (59.6)	1810 (45.6)	291 (40.9)	
NOAC	3247 (22.5)	5 (2.6)	67 (6.7)	378 (12.5)	1230 (22.3)	1328 (33.5)	239 (33.6)	
P2Y12 inhibitor	1605 (11.1)	34 (17.7)	106 (10.6)	336 (11.1)	600 (10.9)	445 (11.2)	84 (11.8)	
Warfarin	369 (2.6)	3 (1.6)	12 (1.2)	41 (1.4)	136 (2.5)	153 (3.9)	24 (3.4)	
Low molecular weight heparin	105 (0.7)	1 (0.5)	5 (0.5)	24 (0.8)	40 (0.7)	28 (0.7)	7 (1.0)	
Any anti-thrombotic therapy	12 530 (86.9)	143 (74.5)	820 (82.0)	2591 (85.7)	4855 (88.0)	3510 (88.4)	611 (85.9)	

Cardiovascular drugs, n (%)								0.0
Beta-blocker	10 313 (71.5)	121 (63.0)	691 (69.1)	2128 (70.4)	4021 (72.9)	2867 (72.2)	485 (68.2)	
Angiotensin-receptor blocker	5467 (37.9)	61 (31.8)	365 (36.5)	1130 (37.4)	2218 (40.2)	1480 (37.3)	213 (30.0)	
Calcium-channel blocker	5071 (35.2)	35 (18.2)	285 (28.5)	1050 (34.7)	2025 (36.7)	1434 (36.1)	242 (34.0)	
ACE inhibitor	4703 (32.6)	79 (41.1)	349 (34.9)	1088 (36.0)	1839 (33.3)	1167 (29.4)	181 (25.5)	
Loop diuretic	3035 (21.1)	12 (6.2)	81 (8.1)	330 (10.9)	1001 (18.1)	1272 (32.0)	339 (47.7)	
Thiazide diuretic	2189 (15.2)	13 (6.8)	121 (12.1)	492 (16.3)	950 (17.2)	547 (13.8)	66 (9.3)	
Mineral receptor antagonist	1874 (13.0)	17 (8.9)	100 (10.0)	352 (11.6)	723 (13.1)	600 (15.1)	82 (11.5)	
Isosorbide mononitrate	2193 (15.2)	11 (5.7)	82 (8.2)	340 (11.2)	780 (14.1)	782 (19.7)	198 (27.8)	
Glyceryl nitrate	1799 (12.5)	23 (12.0)	134 (13.4)	413 (13.7)	645 (11.7)	490 (12.3)	94 (13.2)	
ARNI	374 (2.6)	4 (2.1)	28 (2.8)	80 (2.6)	173 (3.1)	88 (2.2)	1 (0.1)	
Other antihypertensive	361 (2.5)	3 (1.6)	21 (2.1)	66 (2.2)	164 (3.0)	97 (2.4)	10 (1.4)	

Numbers are presented as mean (SD) for continuous variables, and as n (%) for categorical variables.

SD: standard deviation. BP: blood pressure. LDL: low-density lipoprotein. COPD: chronic obstructive pulmonary disease. SGLT2i: sodium–glucose cotransporter 2 inhibitor. DPP-4: dipeptidyl peptidase-4. GLP-1 RA: glucagon-like peptide 1 receptor agonists. PCSK9: proprotein convertase subtilisin/kexin type 9. NOAC: non-vitamin K antagonist oral anticoagulant. ACE: angiotensin converting enzyme. ARNI: angiotensin receptor neprilysin inhibitor.

Supplementary Table S4. Variation among primary health care centres regarding dispensed SGLT2-inhibitors and GLP-1 receptor agonists among patients with ischemic heart disease and diabetes in Region Västra Götaland, Sweden. Multilevel regression models were used to estimate median odds ratios (MOR) with 95% confidence intervals. Continuous variables were modelled using splines.

	**Model 1**		**Model 2**		**Model 3**	
	**MOR**	**95% CI**	**MOR**	**95% CI**	**MOR**	**95% CI**
SGLT2-inhibtor	1.29	1.23–1.36	1.28	1.22–1.35	1.27	1.21–1.34
GLP-1 RA	1.46	1.35–1.59	1.49	1.38–1.63	1.45	1.34–1.58

Model 1: unadjusted. Model 2: adjusted for patient characteristics: age, sex, and comorbidities: hypertension, asthma, and chronic obstructive pulmonary disease. Model 3: adjusted for patient characteristics: age, sex, comorbidities, and primary health care centre characteristics: care need index, adjusted clinical groups, number of enrolled patients, and public/private ownership. Dispensed medications were registered up to 120 days before the index date 1 September 2023. MOR: median odds ratio. CI: confidence interval. SGLT2: sodium–glucose cotransporter 2. GLP-1 RA: glucagon-like-peptide 1 receptor agonists.

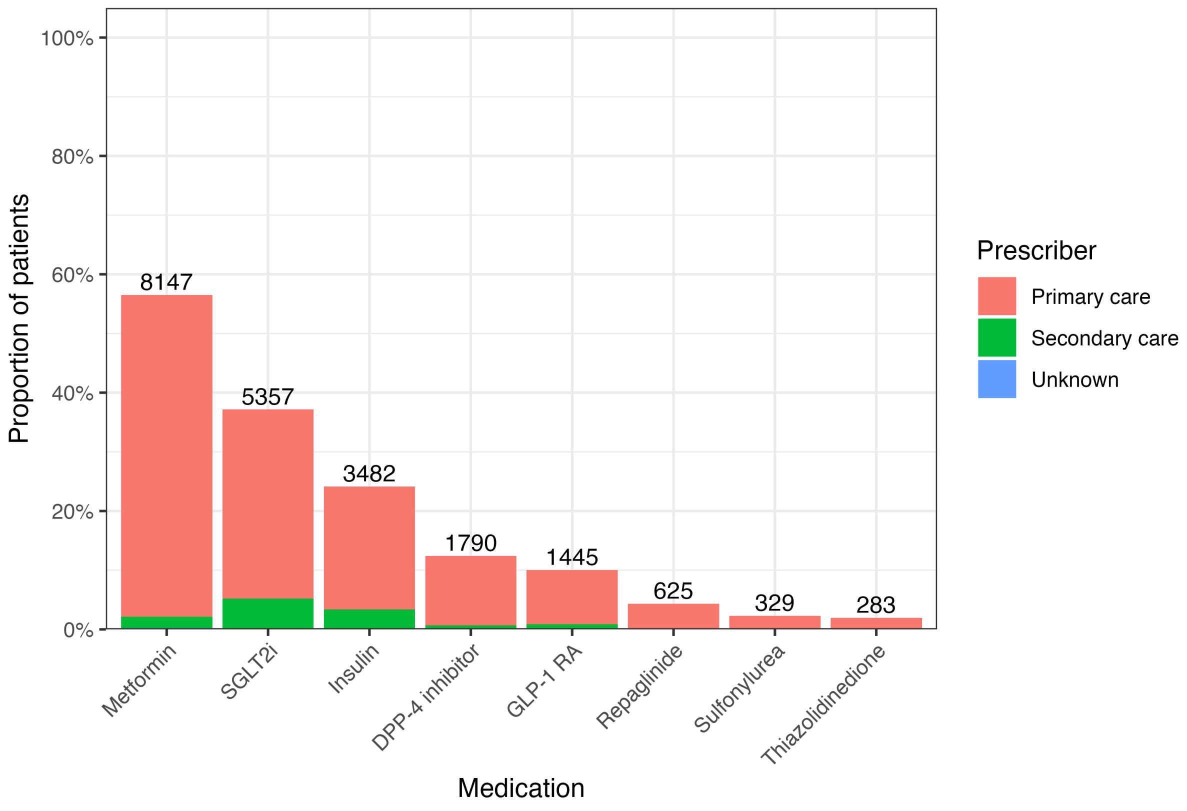

Supplementary Figure S1. Numbers and proportions of patients with dispensed classes of antidiabetic medications within 120 days prior to the index date, stratified by type of prescriber.

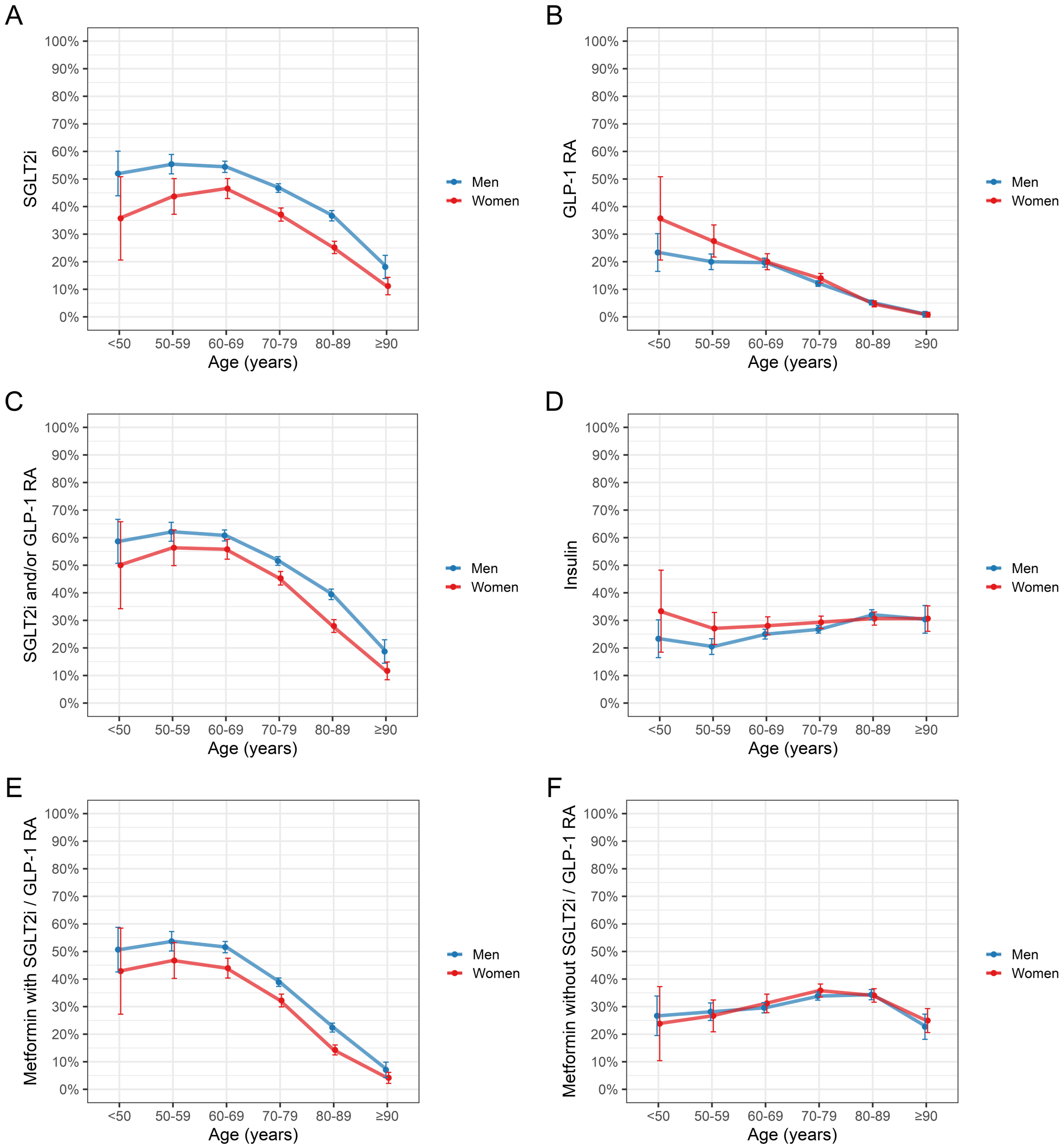

Supplementary Figure S2. Panel figure depicting proportions of patients with dispensed drugs within one year prior to 1 September 2023 according to age and sex. Panel A: SGLT2-inhibitor, B: GLP-1 receptor agonist, C: SGLT2-inhibitor and/or GLP-1 receptor agonist, D: insulin, E: metformin with SGLT2-inhibitor and/or GLP-1 receptor agonist, and F: metformin without SGLT2-inhibitor or GLP-1 receptor agonist. The error bars represent 95% confidence intervals.

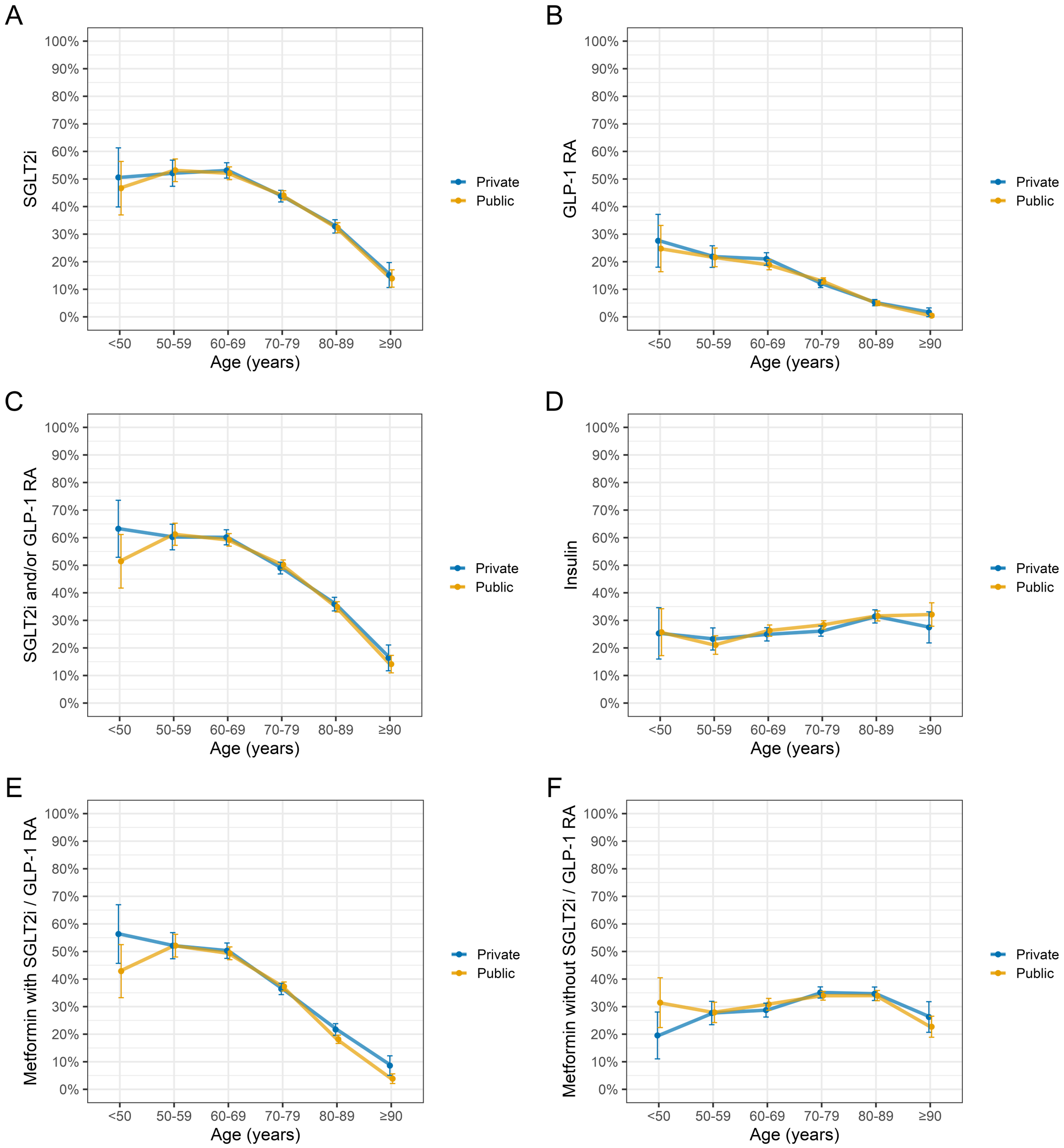

Supplementary Figure S3. Panel figure depicting proportions of patients with dispensed drugs within one year prior to 1 September 2023 according to age and primary health care centre ownership. Panel A: SGLT2-inhibitor, B: GLP-1 receptor agonist, C: SGLT2-inhibitor and/or GLP-1 receptor agonist, D: insulin, E: metformin with SGLT2-inhibitor and/or GLP-1 receptor agonist, and F: metformin without SGLT2-inhibitor or GLP-1 receptor agonist. The error bars represent 95% confidence intervals.

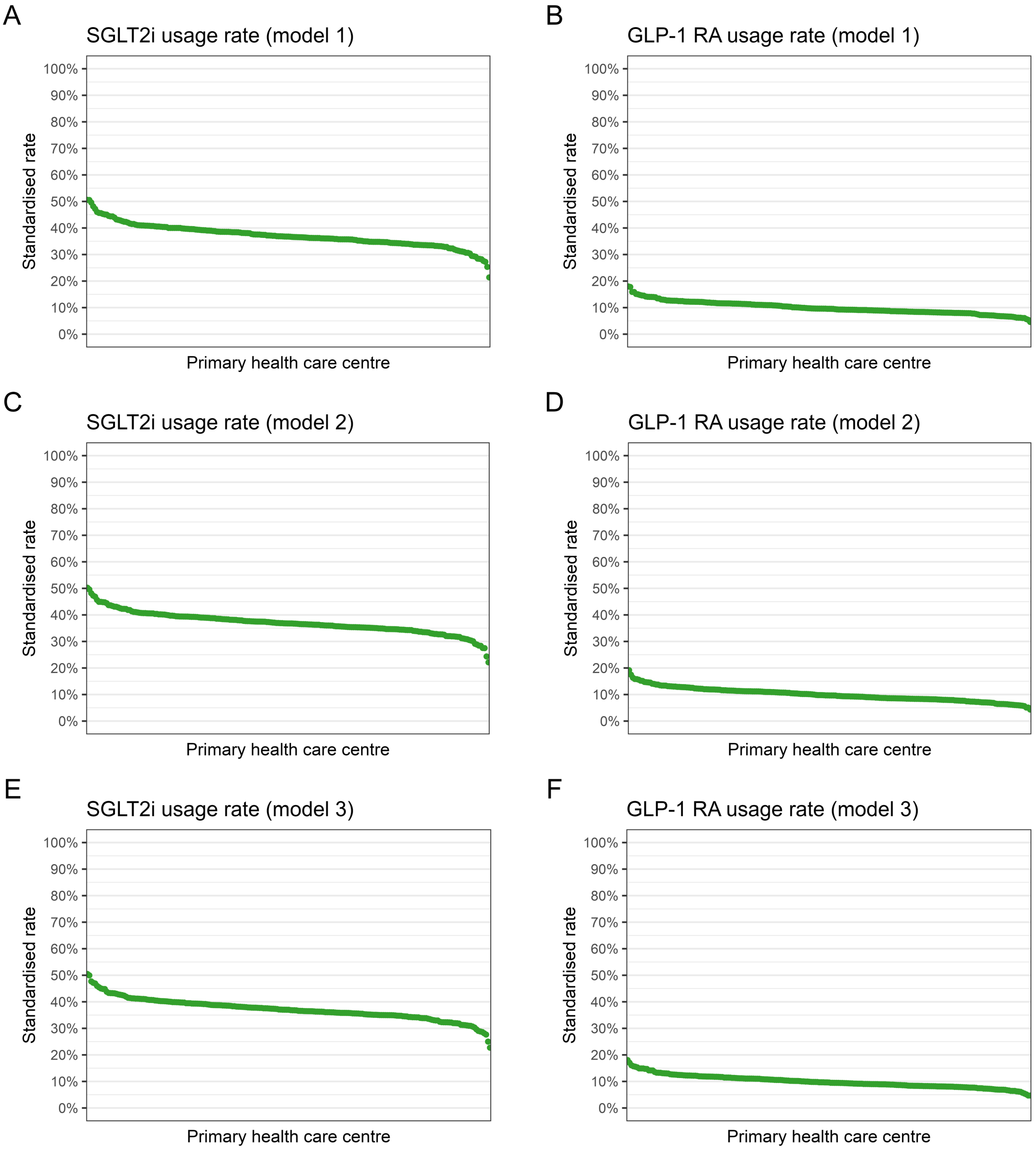

Supplementary Figure S4. Panel figure depicting direct standardised rates of usage of SGLT2i and GLP-1 RA across PHCCs. The rates were obtained from multilevel regression models and standardised to the 2023 patient population in Region Västra Götaland. Model 1: unadjusted rates, that is shrinkage but no standardisation. Model 2: adjusted for age and sex. Model 3: adjusted for patient characteristics: age, sex, comorbidities (hypertension, asthma, and chronic obstructive pulmonary disease), and primary health care centre characteristics: care need index, adjusted clinical groups, number of enrolled patients, and public/private

ownership. These model-based standardised rates are adjusted for the covariates in the models, as specified above, and they exhibit "shrinkage". Multilevel models implicitly penalise deviations from the average PHCC, which reduces the standard error on the PHCC-specific estimates but slightly biases them towards the global average. The absolute amount of bias is larger for PHCCs with fewer observations, and smaller for PHCCs with more observations or with crude rates closer to the global average. This shrinkage reduces the influence of chance outliers.
