# Supplementary material for: Use of SGLT2 inhibitors and GLP-1 receptor agonists in patients with ischaemic heart disease and type 2 diabetes in Swedish primary care: a cross-sectional analysis of regional primary care registry data (QregPV)
Source: BMJ Open. 2026 Feb 2;16(2):e110395. doi: 10.1136/bmjopen-2025-110395 (PMC12878446; doi:10.1136/bmjopen-2025-110395)
Supplement: Supplementary file 2 [file bmjopen-2025-110395.reviewer_comments.pdf]

PEER REVIEW HISTORY

BMJ Open publishes all reviews undertaken for accepted manuscripts. Reviewers are asked to complete a checklist review form and are provided with free text boxes to elaborate on their assessment. These free text comments are reproduced below.

ARTICLE DETAILS

Title (Provisional)

Use of SGLT2 inhibitors and GLP-1 receptor agonists in patients with ischaemic heart disease and type 2 diabetes in Swedish primary care: a cross-sectional analysis of regional primary care registry data (QregPV)

Authors

Andersson, Tobias; Bager, Johan-Emil; Hellgren, Margareta; Åberg, Maria; Mourtzinis, Georgios

---

VERSION 1 - REVIEW

**Reviewer**	**1**
**Name**	**Peasah, Samuel**
**Affiliation**	**UPMC, Health Plan**
**Date**	**11-Dec-2025**
**COI**	**None**

Authors have studied an important topic, well analyzed, but I have few comments for consideration:

1. Authors analyzed use of SGLT2/GLP-1 from a primary care data of patients with type-2 diabetes and ischemic heart disease, based on guideline recommendation of its use as first-line drugs.

2. Please define the index-date (pg. 8 line 6)

3. Page 8 line 7. ‘The proportion of patients who were dispended….’ Please use ‘dispensed’ instead

4. Table 1. Can you explain why more than 10% of the patients are not taking any antidiabetic medications? Is it data issue or were they pre-diabetic?

5. Page 15 line 14. ‘1n 2023, the annual pharmacy cost….’ From who’s perspective? Are you referring to copays by patients? Cost to PHCC? Or both?

6. How does the rate of use of SGLT2/GLP-1 among patients with co-conditions T2D and IHD, compared to those with only T2D nationally?

Reviewer 2

Name Pu, Jun

Affiliation Ren Ji Hospital, School of Medicine, Shanghai Jiao Tong University, Cardiology

Date 04-Jan-2026

COI None

This manuscript investigated the use of sodium-glucose cotransporter 2 inhibitors (SGLT2i) and glucagon-like peptide 1 receptor agonists (GLP-1 RA) among patients with coexisting ischaemic heart disease (IHD) and type 2 diabetes (T2D) in primary care, in relation to European guidelines. This topic is interesting and clinically meaningful, but more clarifications and improvements are still needed.

1. Assessing the utilization of SGLT2i and GLP-1 RA among patients with coexisting IHD and T2D in primary care holds clinical significance. However, the rationale provided for the research background is somewhat inadequate and needs to be better anchored in the previous literature. The clinical necessity and novelty of this investigation should be further clarified.

2. Abstract: the statement “Use declined with age ($p < 0.001$)” (page 2, line 24) fails to specify which medication the term “use” refers to.

3. The definitions and calculation methodologies of two key PHCC-level variables—Adjusted Clinical Group (ACG) and Care Need Index (CNI)—are not specified in the Method Section.

4. Please clarify whether patients with contraindications to SGLT2i or GLP-1 RA (e.g., severe renal insufficiency for SGLT2i, a history of medullary thyroid carcinoma for GLP-1 RA, or any other relevant contraindications) were excluded from the study. If not, the potential impact on the interpretation of medication utilization results should be acknowledged and discussed.

5. The rationale for defaulting diabetes patients in QregPV to T2D should be further justified with specific data (e.g., any reported proportion of T1D patients managed in Swedish primary care). Potential biases caused by this assumption should be discussed in the limitation section.

6. The rationality of defining medication use based on dispensing records within 120 days before the index date needs to be explained. Given that the standard prescription duration in Sweden is 90-100 days, it is necessary to discuss whether the 120-day time window may lead to underestimation or overestimation of long-term medication adherence.

7. The impact of Sweden’s healthcare policies, medical insurance coverage and clinical practice norms on SGLT2i/GLP-1 RA prescription behavior is not sufficiently discussed. These contextual factors are important for interpreting the study findings and for facilitating meaningful comparisons with results from other healthcare settings.

8. Potential selection bias (e.g., under-registration of patients in the database) and information bias (e.g., errors in diagnostic or medication records) should be identified and discussed.

9. Please clarify whether ethical approval was obtained and provide the relevant details.

**Reviewer**	**3**
**Name**	**Yamaji, Kyohei**
**Affiliation**	**Kyoto University Graduate School of Medicine, Division of Cardiology**
**Date**	**07-Jan-2026**
**COI**	**None**

The authors report a large observational cross-sectional study evaluating the use of SGLT2 inhibitors and GLP-1 receptor agonists among patients with ischemic heart disease and type 2 diabetes in primary care. Despite European guideline recommendations, utilization of these agents appears relatively low, with notable inter-practice variation. There was an important evidence-practice gap; however, given the study design, it is not possible to distinguish inappropriate non-use from clinically justified non-prescription such as contraindications, intolerance, and patient preference.

Specific comments

In addition to SGLT2 inhibitors and GLP-1 receptor agonists, other therapies including antiplatelet agents, lipid-lowering therapies, and ACE inhibitors/ARBs are also Class I recommendations in this population. Exploring whether the use of SGLT2 inhibitors and GLP-1 receptor agonists correlates with the prescription of these established guideline-recommended therapies may help to better understand the findings.

Consideration of additional clinical factors influencing treatment eligibility, such as heart failure status and frailty, may further strengthen the interpretation of the adjusted estimates, including the observed sex differences.

The lower use of SGLT2 inhibitors among women could potentially reflect unmeasured clinical factors, such as a higher susceptibility to urinary tract infections, and this possibility may warrant further discussion.

While the adjusted median odds ratios suggest substantial inter-practice variation, interpretation of these findings may be enhanced by acknowledging the lack of available data on practice-level characteristics that could influence prescribing patterns.

VERSION 1 - AUTHOR RESPONSE

Reviewer: 1

Dr. Samuel Peasah, UPMC

Comments to the Authors:
Authors have studied an important topic, well analyzed, but I have few comments for consideration:

Response: We appreciate the reviewer's positive evaluation of the manuscript and the constructive feedback provided.

1. Authors analyzed use of SGLT2/GLP-1 from a primary care data of patients with type-2 diabetes and ischemic heart disease, based on guideline recommendation of its use as first-line drugs.

Response: Thank you for the concise summary of our study, which accurately reflects its aim and clinical context.

2. Please define the index-date (pg. 8 line 6)

Response: The index date was defined earlier in the first sentence of the *Participants and data sources* section. For improved clarity, we have also added this information to the sentence referred to by the reviewer (page 8, line 12):

"…last 120 days before the index date (1 September 2023)."

3. Page 8 line 7. 'The proportion of patients who were dispended….' Please use 'dispensed' instead

Response: Thank you, we have changed this typo (page 8, line 14).

4. Table 1. Can you explain why more than 10% of the patients are not taking any antidiabetic medications? Is it data issue or were they pre-diabetic?

Response: Thank you for this comment. All patients in the study cohort had a recorded diagnosis of T2D. Thus, they were not pre-diabetic. A minority of patients with T2D are managed non-pharmacologically, primarily with lifestyle modifications. This approach is most common among selected elderly patients, for example those with adequate metabolic control without pharmacological treatment or frail patients in whom quality of life is prioritised over strict glycaemic targets. In addition, our analyses are based on dispensed medications, meaning that some patients may have been prescribed antidiabetic treatment but did not fill their prescription. This is consistent with real-world clinical practice and aligns with data from the 2023 annual report of the Swedish National Diabetes Register (reference #10 in Swedish, page 72), which reports that 11.6% of patients with T2D in primary care are treated non-pharmacologically.

5. Page 15 line 14. '1n 2023, the annual pharmacy cost….' From who's perspective? Are you referring to copays by patients? Cost to PHCC? Or both?

Response: Thank you for the comment. For clarification, we have added the following sentence to the last paragraph of the *Results* section (page 15, lines 16–17):

"The cost refers to the combined payment by patients and the reimbursement system."

6. How does the rate of use of SGLT2/GLP-1 among patients with co-conditions T2D and IHD, compared to those with only T2D nationally?

Response: According to data from the Swedish National Diabetes Register, 13% of patients with T2D in Sweden were prescribed a GLP-1 RA and 19% an SGLT2 inhibitor in 2023. However, prescribing rates stratified by the presence of IHD are not provided, and a direct national comparison between patients with T2D with and without IHD is therefore not available. We have added this sentence to the *Discussion* in the second paragraph of the *Comparison to previous studies* section (page 17, lines 10–13):

"The use of SGLT2i and GLP-1 RA in our study can be viewed in relation to the general T2D population in Sweden, where, according to the Swedish National Diabetes Register, 19% of patients were prescribed an SGLT2i and 13% a GLP-1 RA in 2023.[20]"

Reviewer: 2

Dr. Jun Pu, Ren Ji Hospital, School of Medicine, Shanghai Jiao Tong University

Comments to the Authors:
This manuscript investigated the use of sodium-glucose cotransporter 2 inhibitors (SGLT2i) and glucagon-like peptide 1 receptor agonists (GLP-1 RA) among patients with coexisting

ischaemic heart disease (IHD) and type 2 diabetes (T2D) in primary care, in relation to European guidelines. This topic is interesting and clinically meaningful, but more clarifications and improvements are still needed.

Response: Thank you for the overall positive assessment and the constructive comments that helped improve our manuscript.

1. Assessing the utilization of SGLT2i and GLP-1 RA among patients with coexisting IHD and T2D in primary care holds clinical significance. However, the rationale provided for the research background is somewhat inadequate and needs to be better anchored in the previous literature. The clinical necessity and novelty of this investigation should be further clarified.

Response: Thank you for this important notice. To strengthen the rationale of the study we have added a reference and expanded the *Introduction* by adding/revising these sentences in the third paragraph (page 5, lines 16–20):

"Low utilization of SGLT2i and GLP-1 RA has been reported among patients in primary care with IHD and newly diagnosed T2D.[4] However, less is known about patients with a heterogeneous duration of disease. Moreover, little is known about the usage of SGLT2i and GLP-1 RA in primary care among patients with IHD and T2D, or how it varies between primary healthcare centres (PHCCs) and by public vs. private ownership."

To further clarify the necessity of the study we have added this sentence to the third paragraph of the *Introduction* (page 6, lines 2–4):

"Given the cardiovascular risk reduction associated with SGLT2i and GLP-1 RA, insufficient implementation may represent a missed opportunity for secondary prevention in a large and high-risk patient population."

2. Abstract: the statement "Use declined with age ($p < 0.001$)" (page 2, line 24) fails to specify which medication the term "use" refers to.

Response: Thank you for pointing out this unclear sentence. We have revised it to:

"Use of SGLT2i and GLP-1 RA declined with age ($p < 0.001$)."

3. The definitions and calculation methodologies of two key PHCC-level variables—Adjusted Clinical Group (ACG) and Care Need Index (CNI)—are not specified in the Method Section.

Response: Thank you for the comment. In addition to the references explaining ACG and CNI we have added this brief explanation in the *Participants and data sources* section (page 7, lines 21–25):

“ACG is a method for describing patient case-mix based on age, sex, and registered diagnoses to predict health care utilization. CNI is based on sociodemographic factors reflecting social deprivation (elderly persons living alone, foreign born individuals, unemployed individuals, single parents, high residential mobility, individuals with low educational status, and children under the age of 5).”

4. Please clarify whether patients with contraindications to SGLT2i or GLP-1 RA (e.g., severe renal insufficiency for SGLT2i, a history of medullary thyroid carcinoma for GLP-1 RA, or any other relevant contraindications) were excluded from the study. If not, the potential impact on the interpretation of medication utilization results should be acknowledged and discussed.

Response: Thank you for the comment. We have acknowledged the lack of data on contraindications by adding the following sentence to the *Strengths and limitations* section (page 21, lines 20–21):

“We had no data on frailty or contraindications to SGLT2i or GLP-1 RA, which may partly affect the estimation of their use.”

5. The rationale for defaulting diabetes patients in QregPV to T2D should be further justified with specific data (e.g., any reported proportion of T1D patients managed in Swedish primary care). Potential biases caused by this assumption should be discussed in the limitation section.

Response: Thank you for this comment. We have added a reference to the 2023 annual report from the Swedish National Diabetes Register to justify defaulting patients with diabetes in QregPV to T2D and have revised the following sentence in the *Participants and data sources* section from:

“As patients with type 1 diabetes are not primarily managed in Swedish primary care, individuals with a recorded diagnosis of diabetes in QregPV are, in practice, considered to have T2D and are referred to as such throughout this study.”

To (page 7, lines 13–17):

“As patients with type 1 diabetes are not primarily managed in primary care and 98% of patients with diabetes in Swedish primary care reported to the National Diabetes Register are

classified as having T2D,[10] individuals with a recorded diagnosis of diabetes in QregPV are, in practice, considered to have T2D and are referred to as such throughout this study."

We have also added the following sentence in the *Strengths and limitations* section of the *Discussion (page 21, lines 24–25 and page 22 lines 1–3)*:

"We assumed that patients with diabetes in QregPV have T2D, as patients with type 1 diabetes are not primarily managed in primary care. However, we cannot rule out that a small proportion of patients with type 1 diabetes may have been included in our study. We do not believe that this has had any significant impact on our results."

6. The rationality of defining medication use based on dispensing records within 120 days before the index date needs to be explained. Given that the standard prescription duration in Sweden is 90-100 days, it is necessary to discuss whether the 120-day time window may lead to underestimation or overestimation of long-term medication adherence.

Response: Thank you for the comment. Our choice of using a 120-day dispensation window corresponds approximately to the widely used adherence standard of an 80% proportion of days covered, given the standard prescription duration of 90–100 days in Sweden. In addition to the conducted sensitivity analyses presented in Supplementary Figure S2 we have revised the last sentence in the *Antidiabetic therapy* section of the *Results* by adding the overall one-year dispensation rates of SGLT2i and GLP-1 RA (page 13, lines 9–11):

"Sensitivity analyses of dispensed medications up to one year prior the index date showed similar dispensation patterns although with higher absolute numbers (SGLT2i 41.8% and GLP-1 RA 12.3%), Supplementary Figure S2."

We believe the one-year dispensation rates in practice can be seen as an upper limit of the use of SGLT2i and GLP-1 RA in the study population. We have expanded the discussion by adding two sentences in the *Strengths and limitations* section (page 22, lines 3–7):

"Our use of dispensation records within 120 days before the index date may have overestimated treatment use, as some patients may have discontinued the medication after dispensation. Underestimation is also possible. However, we believe that the one-year dispensation rates, which were slightly higher, indicate a reasonable upper bound for treatment use."

7. The impact of Sweden's healthcare policies, medical insurance coverage and clinical practice norms on SGLT2i/GLP-1 RA prescription behavior is not sufficiently discussed. These contextual factors are important for interpreting the study findings and for facilitating meaningful comparisons with results from other healthcare settings.

Response: Thank you for the comment. We have expanded the discussion regarding the specific Swedish clinical context by adding two sentences in the *Clinical implications* section of the *Discussion* (page 19, lines 16–20):

"It may also vary within countries; for example, in Sweden, where the cost of medications prescribed at PHCCs in different counties may be covered either by the individual PHCC or at the county level. As GLP-1 RA are significantly more expensive than SGLT2i, the financial obstacle to prescribing them is likely even greater."

8. Potential selection bias (e.g., under-registration of patients in the database) and information bias (e.g., errors in diagnostic or medication records) should be identified and discussed.

Response: Thank you for the comment. We have added these two sentences to the *Strengths and limitations* section of the *Discussion* to further discuss potential selection bias in QregPV (page 21, lines 3–6):

"However, eligible patients without a recorded diagnosis of T2D or IHD are not reported to QregPV. We consider the risk of resulting selection bias to be small given the relatively large number of patients included in the study."

Regarding medication records, we have clarified in the following sentence that the coverage of dispensed medications is complete (page 21, lines 6–8):

"Second, we use complete individual-level data on dispensed medications, which more accurately reflect patients' actual use compared to prescribed medication data."

9. Please clarify whether ethical approval was obtained and provide the relevant details.

Response: Thank you for pointing this out. In addition to information already provided in the submission system, we have now added an *Ethics approval* statement to the manuscript (page 24, lines 13–16).

Reviewer: 3

Dr. Kyohei Yamaji, Kyoto University Graduate School of Medicine

Comments to the Authors:
1. The authors report a large observational cross-sectional study evaluating the use of SGLT2 inhibitors and GLP-1 receptor agonists among patients with ischemic heart disease and type 2 diabetes in primary care. Despite European guideline recommendations, utilization of these agents appears relatively low, with notable inter-practice variation. There was an important

evidence-practice gap; however, given the study design, it is not possible to distinguish inappropriate non-use from clinically justified non-prescription such as contraindications, intolerance, and patient preference.

Response: Thank you for this comment. We agree that our study has some limitations as acknowledged in the *Strengths and limitations* section. We have expanded on this further by adding the following sentence (page 21, lines 18–19):

"Given the study design, we could not distinguish inappropriate non-use from clinically justified non-prescription such as intolerance and patient preference."

Specific comments
2. In addition to SGLT2 inhibitors and GLP-1 receptor agonists, other therapies including antiplatelet agents, lipid-lowering therapies, and ACE inhibitors/ARBs are also Class I recommendations in this population. Exploring whether the use of SGLT2 inhibitors and GLP-1 receptor agonists correlates with the prescription of these established guideline-recommended therapies may help to better understand the findings.

Response: Thank you for the comment. In table 1, we present the corresponding proportions of patients with any lipid-lowering therapy (82.8%), any anti-thrombotic therapy (86.9%), and ACE inhibitors (32.6%)/ARB (37.9%). Thus, those class 1 recommendation medications reach significantly higher levels of use than SGLT2i and GLP-1 RA.

3. Consideration of additional clinical factors influencing treatment eligibility, such as heart failure status and frailty, may further strengthen the interpretation of the adjusted estimates, including the observed sex differences.

Response: Thank you for this very important remark. Unfortunately, we do not have data on frailty or comorbidities such as heart failure and chronic kidney disease. However, as a proxy for frailty we see that the use of SGLT2i and GLP-1 RA is lowest among the elderly (Figure 2). We acknowledge these limitations and have added this sentence in the *Strengths and limitations* section (page 21, lines 20–21):

"We had no data on frailty or contraindications to SGLT2i or GLP-1 RA, which may partly affect the estimation of their use."

4. The lower use of SGLT2 inhibitors among women could potentially reflect unmeasured clinical factors, such as a higher susceptibility to urinary tract infections, and this possibility may warrant further discussion.

Response: Thank you for this thoughtful comment. We have added this sentence accordingly in the *Clinical implications* section of the *Discussion* (page 19, lines 5–7):

"Higher susceptibility to urinary tract infections among women, with potential subsequent discontinuation of SGLT2i, may possibly be reflected in the lower use of SGLT2i observed in women in our study."

5. While the adjusted median odds ratios suggest substantial inter-practice variation, interpretation of these findings may be enhanced by acknowledging the lack of available data on practice-level characteristics that could influence prescribing patterns.

Response: Thank you for the comment. Although we have various PHCC data such as ACG, CNI, number of enrolled patients, and whether public or private ownership, we also lack some potential practice level characteristics that could influence prescribing patterns. We have acknowledged this in the *Strengths and limitations* section (page 21, lines 15–18):

"Furthermore, in the calculation of the median odds ratio to reflect inter-practice variation, we did not have access to individual health care provider continuity or characteristics, such as age, sex, and level of experience, which may influence prescribing patterns."

VERSION 2 - REVIEW

**Reviewer**	**1**
**Name**	**Peasah, Samuel**
**Affiliation**	**UPMC, Health Plan**
**Date**	**23-Jan-2026**
**COI**	

Authors have addressed reviewers concerns adequately. I recommend the manuscript to be accepted.
